# Supplementary material for: What does it take to learn the rules of RNA base pairing? A lot less than you may think
Source: bioRxiv. 2025 Aug 2:2025.07.31.668042. Preprint. [Version 1] doi: 10.1101/2025.07.31.668042 (PMC12324431; doi:10.1101/2025.07.31.668042)
Supplement: Supplement 1 [file media-1.gz › supplemental_material/src/rscape_v2.6.0.tar.gz!/rscape_v2.6.0/lib/infernal/Manuscripts/filter_thresholds/main.pdf]

Filter sensitivity targeting for RNA similarity searches

Eric P. Nawrocki and Sean R. Eddy
HHMI Janelia Farm Research Campus
19700 Helix Drive
Ashburn VA 20147
http://selab.janelia.org/

May 25, 2009

Motivation:

Covariance models (CMs) are profile probabilistic models for RNA similarity search that score both sequence and secondary structure conservation. The practical application of CMs has been limited by the high computational complexity of their dynamic programming search algorithms.

Results:

We present a filtering technique for CM searches called filter sensitivity targeting (FST) that determines filter thresholds to maximize speed while maintaining a target level of sensitivity. When applied to HMM filtering, FST speeds up CM searches by about 25-fold while sacrificing very little sensitivity on our benchmark.

Availability:

The source code for INFERNAL 1.01, which includes FST, and the benchmark are downloadable from `http://infernal.janelia.org`. INFERNAL is freely licensed under the GNU GPLv3 and should be portable to any POSIX-compliant operating system, including Linux and Mac OS/X.

Contact: `{nawrockie,eddys}@janelia.hhmi.org`

Introduction

Computational methods for searching for homologous RNAs in sequence databases benefit from scoring both primary sequence and RNA secondary structure conservation. Many tools have been developed which take different approaches to how RNA sequence and secondary structure constraints should be integrated and scored. Some tools implement specialized rules for a specific RNA family, such as tRNAs [1, 2], snoRNAs [3, 4], microRNAs [5, 6], or SRP RNAs [5, 6]. Some approaches use pattern matching methods and expertly designed query patterns [7]. The most general approaches take as input any RNA (or RNA multiple alignment), and construct an appropriate statistical scoring system (often called a profile) that allows quantitative ranking of putative homologs in a target sequence database [8–10].

Stochastic context-free grammars (SCFGs) provide a natural statistical framework for combining sequence and (non-pseudoknotted) secondary structure conservation information in a single consistent scoring system [11–14]. A particular formulation of profile SCFGs, the covariance model (CM), was developed specifically for the RNA similarity search problem. A CM is a probabilistic model built from a single RNA sequence or multiple alignment with consensus secondary structure annotation marking which positions of the alignment are single stranded and which are base paired. A CM is organized as a binary tree of different types of states that model different sequence and structural features of the RNA family they model. For example, there are different state types for single stranded residues, basepairs, insertions, and deletions. The states include position specific scores for the four possible residues at single stranded positions, the sixteen possible base pairs at paired positions, and for insertions and deletions relative to the consensus sequence and structure. These scores are log-odds scores derived from the observed counts of residues, base pairs, insertions and deletions in the input alignment, combined with prior information derived from structural ribosomal RNA alignments. CM construction and parameterization has been described in more detail elsewhere [12, 15**?** , 16].

A major use of CMs is within the RFAM database, which contains alignments and CMs for over a thousand RNA families [17]. RFAM uses CM similarity searches to annotate RNAs in the RFAMSEQ, a 120 Gb nucleotide database derived from the EMBL database [**?**]. A recent independent benchmark of RNA similarity search found CM-based methods to be the most sensitive, but also the slowest, of the several that were tested [18]. The high computational complexity and resulting slow speed of CM dynamic programming search algorithms have been a large obstacle to their practical application.

Two complementary approaches have been taken to mitigate this high computational cost. The first is

to accelerate the CM CYK similarity search dynamic programming algorithm. We introduced a banded variant of CYK that reduces the average case time complexity from $LN^{2.4}$ to $LN^{1.3}$, for a query of length N residues (or consensus alignment columns) and a target database of length L, at a small cost to sensitivity [16]. The second approach is to reduce the search space (decrease L) by using a filter to quickly prune away regions of the database that are unlikely to contain high scoring hits to the CM. The CYK algorithm is then only used on the surviving fraction of the database.

Several filtering techniques have been developed for CMs. RFAM uses a BLAST-based filter on the RFAMSEQ target database prior to searching with CMs. All sequences from the CM training alignment are used as queries, any target subsequence scored with an E-value less than 1000 to any query survives the filter and is searched with the CM. Weinberg and Ruzzo introduced two types of HMM filters, "rigorous filter" HMMs ([19]) and "ML (maximum likelihood) HMMs" [20]. Rigorous filter HMMs are parameterized to provably allow all target subsequences that score better than a preset CM score threshold to survive. ML HMMs are built to be as similar as possible to the CM. ML HMM filtering aims to prune away 99% of the target database by setting the filter survival threshold so that a predicted 1% of the database will score better than it. Zhang et al. [21] have described keyword based techniques for filtering that require a database subsequence contain at least one of a pre-generated list of keywords to survive the filter. Their technique is similar to HMM rigorous filters in that all hits above a preset CM score threshold are guaranteed to survive.

An important tradeoff exists between the acceleration gained and the sensitivity lost from using a filter. Acceleration is dependent on the speed of the filtering technique and the fraction of the database that survives the filter. The sensitivity loss depends on the ability of the filter to recognize (and not prune away) possible high scoring CM hits. The aforementioned filtering strategies prioritize speed versus sensitivity differently. Weinberg/Ruzzo ML HMM filtering is designed for speed by pruning away a target fraction of the database. Alternatively, filter survival thresholds can be set to achieve a target sensitivity. For example, with Weinberg/Ruzzo rigorous filter HMMs and Zhang/Bafna keyword filters, the survival threshold is set so that 100% of the target subsequences that score better than a preset CM threshold will survive, regardless of the resulting survival fraction.

Approach

We propose a technique for determining filter survival thresholds that will achieve any target level of sensitivity. This technique, which we call filter sensitivity targeting (FST), is similar to Weinberg/Ruzzo's

rigorous filters and Zhang/Bafna's keyword filters in that it prioritizes sensitivity over speed, but differs in that it does not provably sacrifice zero sensitivity. A potential advantage of FST over the other methods is that it may be faster (by pruning away more of the database) while sacrificing an acceptably small amount of sensitivity. First, we describe the general FST procedure, which can be applied to any type of filter for any type of similarity search method. Then we focus on the application of FST to HMM filtering for RNA similarity searches with CMs.

Determining filter survival thresholds by filter sensitivity targeting (FST)

Filtered database searches involve two search algorithms, which we will refer to as the *filter* algorithm and the *final* algorithm. The database is scored first with the filter algorithm, and surviving subsequences, or hits, are rescored with the final algorithm. We define the sensitivity F of a filter as the fraction of database hits that survive the filter (score above a filter survival score threshold T) that a non-filtered search with only the final algorithm would report (score above a reporting score threshold C). FST is a procedure for estimating the appropriate T to use to achieve F sensitivity for a search using threshold C. The only required input of the procedure is the desired F, and a set of N test sequences. There are three main steps to FST:

1. Score N test sequences using both the filter and final algorithms.

2. Create two lists of the sequences. Sort list 1 by increasing final score. Sort list 2 by increasing filter score.

3. For sequence $i = 1$ to N, with final score C_i, in list 1:

   - Prune list 2 to only include the $(N - i + 1)$ sequences with final score $>= C_i$
   - Set the filter threshold T_i equal to the $(F * (N - i + 1))$ ranked filter score from pruned list 2.
   - Save (T_i, C_i) as a filter survival threshold/final reporting threshold score pair.

When finished, each (T, C) pair indicates a filter survival score threshold T to use when searching with final reporting threshold C to theoreatically achieve filter sensitivity F. If the test sequences are a representative sample of the real target homologous sequences, then in the limit of very large N and infinite database searching, using (T, C) in this way will achieve sensitivity F. In other words, the larger and more representative of real homologs the set of test sequences is, the more accurate, and consequently useful, the FST approach is.

A caveat to the procedure is that in step 3, as i approaches N, $(N - i)$, the number of test sequences used to determine T_i, approaches zero. Because the accuracy of FST depends on the number of test sequences being large, it's reasonable to set a max on i, in practice we use $0.9 * N$, so that at least $0.1 * N$ test sequences are used to determine all (T, C) pairs.

Figure 1 shows data for the FST procedure for three anecdotal RNA families using $N = 10,000$ and $F = 0.993$. Each small point represents a sequence, with x-coordinate equal to filter score and y-coordinate equal to final score. The larger points are a subset of the (T, C) pairs. For each (T, C) point, the fraction of small points with $y > C$ that have $x < T$ is $1 - F = 0.007$, these represent the 0.7% of sequences that a non-filtered search would find that a filtered search will not find.

Source of test sequences

An important question is: how do we obtain the test sequences? One approach is to use known examples of homologs. Weinberg and Ruzzo essentially suggested a special case of the FST strategy to define thresholds for ML HMM filters for CM searches by using the RFAM "seed" sequences as the N sequences and requiring an F of 1.0. (They ultimately decided on using filter survival thresholds that would eliminate 99% of the target database as their thresholding strategy.) The seed sequences are the sequences in the RFAM structural alignment used to build the CM. Alternatively, the RFAM "full" sequences could be used, which are all the sequences that score above an expertly curated score threshold (chosen as the score of the highest scoring obvious false positive) in a BLAST filtered CM search of the RFAMSEQ database.

For structural RNAs, there are two drawbacks to using known homologs as the N test sequences. First, the number of known homologs is usually small. The median number of seed plus full sequences per RNA family in RFAM release 9.1 (by far the largest public database of RNAs) is 50, with 100 or more sequences in 30% of the families, and 1000 or more sequences in only 6%. This is problematic because the accuracy of FST depends on N being large. Secondly, known homologs are unlikely to be a representative sample of the sequences the CM would classify as homologous with stastically significant scores. Alignments of the seed sequences are used to build and paramterize the models themselves, and as a result those sequences are a biased sample of very high scoring sequences. The full sequences have been detected using a BLAST filter and, presumably, are also a biased, high scoring sample (although it is impossible to be certain without doing a prohibitively expensive non-filtered CM search for comparison). CM parameterization has recently been significantly improved for remote homology detection [16], with the adaptation of informative mixture

Dirichlet priors and entropy weighting from profile HMM implementations. In order for a FST calibrated filter to maintain that increased sensitivity, the test sequences must include lower scoring, but still statistically significant, remotely homologous sequences.

An alternative source of the test sequences is to take advantage of the generative capacity of CMs as probabilistic models and sample the test sequences directly from the model. This approach addresses the requirements of our strategy. N can be large because sampling is fast and infinitely repeatable, and sampling draws sequences from the CM's own probability distribution, which is exactly the distribution of homologs the CM is modelling. Figure 2 illustrates the difference in the CM score distributions of random sequences (solid lines), known (RFAM seed *and* full sequences, dotted lines), and sampled sequences (dashed lines) for three anecdotal RNA families: tRNA, 5S rRNA, and SRP RNA. In all three cases, the known sequences are biased towards high scores relative to the sampled sequences.

Scoring and sampling sequences

CM similarity search algorithms assign a bit score to a target database subsequence. The bit score B is a log odds score: $B = \log_2 \frac{P(\text{seq}|\text{CM})}{P(\text{seq}|\text{null})}$. $P(\text{seq}|\text{CM})$ is the probability of a target subsequence according to the CM. The *Inside* dynamic programming algorithm calculates this value by summing the probability of all possible paths π through the model that generate the subsequence, that is: $P(\text{seq}|\text{CM}) = \sum_{\pi} P(\text{seq}, \pi|\text{CM})$. [14]. $P(\text{seq}|\text{null})$ is the probability of the target sequence given a "null hypothesis" model of the statistics of random sequence. The null model is a simple one-state hidden Markov model (HMM) that says that random sequences are i.i.d. sequences with a specific residue composition, which is equiprobable across the four RNA nucleotides (0.25 each). Therefore the null model score is calculated as: $P(\text{seq}|\text{null}) = 0.25^L$ for a sequence of length L. Because this null model score depends only on the length of the target sequence, and not the sequence itself, B increases monotonically with $P(\text{seq}, \pi|\text{CM})$ for a constant L. As the probability that a sequence was generated from the CM increases, so does it's score. This suggests that sampling from the disbribution defined by: $P(\text{seq}, \pi|\text{CM})$ should yield high scoring sequences. This is confirmed anecdotally for three families in Figure 2 for which the scores of the vast majority of sampled sequences are significantly better than random.

Sampling a sequence from a CM is a recursive procedure that begins at the root state and samples a tree of states, called a parsetree, and sequence residues, until all branches of the tree terminate at end states. The emitted sequence associated with a parsetree is generated from outside to inside (as opposed to from left to

right from an HMM). When singlet or basepair emitting states are visited a single residue or basepair residue, respectively, is sampled from the state's emission probability distribution. If the emitting state is a singlet left-emitting state, the sampled residue is appended on the right (3') to the left half of the nascent sequence. Conversely, if the emitting state is a singlet right-emitting state, the sampled residue is appended on the left (5') to the right half of the sequence. Basepair states behave as both a left-emitting and right-emitting state, emitting one residue to the left and one to the right. Finally, when bifurcation states are visited, two new paths are created, one beginning at the left child state and one at the right child state, and each of these paths is continued until an end state is reached. The time complexity of the sampling procedure is $O(N)$ time for a CM of N states. Roughly 10,000 paths can be sampled from average sized CM per second.

CMs can be locally or globally configured [22**?**]. In global mode, the only way to enter and exit the model is through the root state and end states, respectively. In local mode, begins and ends are possible from any internal node of the model. Further, when a local end takes place, a special insert state is visited that can emit additional sequence. Local ends allow CMs to tolerate insertions or deletions of entire substructures, increasing sensitivity for remote homology detection in some cases.

Practical limits on filter survival thresholds

When finding survival threshold T, FST prioritizes sensitivity over speed by ignoring the effect using T will have on the running time of a filtered search. We can further prioritize sensitivity over speed by enforcing a maximally useful filter survival threshold T_{max}, and to use $min(T, T_{max})$ for any FST derived T. This can only increase the sensitivity of the filter (because $T_{max} < T$) at a cost to speed. However, T_{max} can be chosen so that the effect on the total running time is negligible.

The running time (t) of a filtered search is the time required to run the filter on the full target (t_f) plus the time required to run the final algorithm on the full target (t_m) multiplied by the fraction that survives the filter (S). That is, $t = t_f + S * t_m$. The survival fraction S is controlled by the survival threshold T: as T increases, S decreases, and vice versa. Because t is directly affected by S, a reasonable way to enforce a T_{max} is to use a single query independent S_{min}, and converting it to a T_{max} for each query. This requires a way of converting between S and T, which is straightforward if E-values are available: $S = \frac{EL}{Z}$, where E is the E-value for T using the filter scoring algorithm, Z is the database size, and L is the average length of a surviving fraction of the database from the filter. The appropriate choice of S_{min} is likely to be highly dependent on the ratio of running times of the filter and final scoring algorithms. We investigate reasonable

S_{min} values to use for HMM filtered CM searches based on empirical performance in a benchmark below.

Using the banded CYK algorithm as a second filter

In some cases, using two filters in succession, or *chain* filtering, can compound the resulting acceleration without sacrificing sensitivity [**?**]. Previously, we developed a banded version of the CYK dynamic programming (DP) algorithm for CM similarity search called query-dependent banding (QDB) [16] that precalculates regions of the DP lattice that have negligible probability and ignores those regions during the DP recursion for greater speed. In our benchmarks, QDB offered about a four-fold speedup at a small cost to sensitivity. This work on filtering led us to reevaluate the primary use of QDB CYK, testing it as a filter for the more sensitive and time consuming Inside algorithm, instead of as a standalone, final algorithm for similarity search. Rather than use FST to determine thresholds for CYK filtering, we tried a simpler, query-independent strategy of setting the filter E-value threshold as 100 times the final algorithm threshold. We discuss our results below.

Implementation

FST for CM similarity searches has been implemented in INFERNAL version 1.01 [23]. The filtering algorithm is the HMM Forward algorithm with a reimplementation of Weinberg/Ruzzo's ML HMMs [20]. FST thresholds are determined for two different main algorithms, the CYK and Inside CM search algorithms. INFERNAL also implements approximate E-values for HMM Forward and CM CYK and Inside scores. E-values are integral to the FST implementation because they allow a predicted survival fraction S to be calculated from a bit score T. FST is executed using N sampled sequences for a single target sensitivity, F, by INFERNAL's `cmcalibrate` program for both local and globally configured CMs. By default, $N = 10,000$ and $F = 0.993$ but both values can be changed by the user. The resulting pairs of survival thresholds T and reporting thresholds C are stored in the CM save file and read by the `cmsearch` program when a database search is executed. (To avoid storing $N = 10,000$ points, a representative set of a few hundred (T, C) pairs is saved in which no two C values C_1, C_2 ($C_1 < C_2$) with E-values E_1 and E_2 ($E_1 > E_2$) follow $E_2 - E_1 < (0.1 * E_1)$.) For a search with final algorithm CYK or Inside with reporting threshold C', T from the CYK/Inside (T, C) pair in the CM file with the maximum $C < C'$ is selected and T is set as the filter surviving threshold. The search proceeds by scanning each target sequence in the database with the filter. For any subsequence $i..j$ that scores above T, the subsequence $j - W + 1..i + W - 1$ is flagged

as a surviving subsequence. (W is the maximum hit length defined as $dmax(0)$ from the band calculation algorithm using $\beta = 10^{-7}$ [16]a). If a second round of filtering is used (as discussed below), it is used in the same manner, but only on the surviving subsequences from the first filter. The final algorithm is used to rescore subsequences that survive all filtering stages. The complete INFERNAL ANSI C source code is included in the Supplementary Material.

Evaluation

To measure the effect of FST and CYK filtering methods on the speed, sensitivity and specificity of RNA similarity searches, we used an improved version of our internal RFAM-based benchmark [16, 23]. Briefly, this benchmark was constructed as follows. The sequences of the seed alignments of 503 RFAM (release 7) families were single linkage clustered by pairwise sequence identity, and separated into two clusters such that no sequence in one cluster is more than 60% identical to any sequence in the other. The larger of the two clusters was assigned as the query (preserving their original RFAM alignment and structure annotation), and the sequences in the smaller cluster were assigned as true positives in a test set. We required a minimum of five sequences in the query alignment. 51 RFAM families met these criteria, yielding 450 test sequences which were embedded at random positions in a 10 Mb "pseudogenome". Previously we generated the pseudogenome sequence from a uniform residue frequency distribution [16]. Because base composition biases in the target sequence database cause the most serious problems in separating significant CM hits from noise, we generated a more realistic pseudogenome sequence using a 15-state fully connected hidden Markov model (HMM) trained by Baum-Welch expectation maximization [14] on genome sequence data from a wide variety of species. Each of the 51 query alignments was used to build a CM and search the pseudogenome in local mode, a single list of all hits for all families were collected and ranked, and true and false hits were defined (as described in Nawrocki and Eddy [16]).

The minimum error rate (MER) ("equivalence score") [24] was used as a measure of benchmark performance. The MER score is defined as the minimum sum of the false positives (negative hits above the threshold) and false negatives (true test sequences which have no positive hit above the threshold), at all possible choices of score threshold in the ranked list of all hits from the 51 searches. The MER score is a combined measure of sensitivity and specificity, where a lower MER score is better. We calculate two kinds of MER scores. For a *family-specific* MER score, we choose a different optimal threshold in each of the 51 ranked lists, and for a *summary* MER score, we choose a single optimal threshold in the master

list of all hits. The summary MER score reflects the performance level for a large scale analysis of many families because it demands a single query-independent E-value reporting threshold for significance. The family-specific MER score indicates the performance that could be achieved with manual inspection and curation of the hits in each family to determine family specific E-value thresholds.

Using this benchmark, we addressed several questions about the performance of FST calibrated HMM filtering and CYK filters.

First, we had to determine the most sensitive CM search strategy irrespective of speed so that we had a best-case performance against which to judge the filtered searches. We tested the Inside and CYK algorithms, both with and without query-dependent bands (QDBs). For the banded runs we used a $\beta = 10^{-15}$ tail loss probability for QDB calculation that previous work has indicated sacrifices essentially zero sensitivity [16]. As shown in Table 1, using the banded Inside algorithm resulted in the lowest summary and family specific MER of the four methods tested (rows 1-4 in Table 1). Interestingly, banded Inside outperforms non-banded Inside (row 1 in Table **??**); this is because enforcement of the bands eliminates about a dozen high scoring alse positive hits that drive up the MERs. This result led us to use banded Inside with $\beta = 10^{-15}$ as the final (post-filtering) search strategy when benchmarking filtered search strategies.

Next, we addressed FST parameterization. What is the best value to use for the F parameter, which specifies the fraction of sequences allowed below the filter score threshold? The black solid points in Figure 3 shows the benchmark running time of FST calibrated HMM filtered searches versus MER for different values of F. The choice of F is a tradeoff of accuracy for speed. We chose a default of $F = 0.993$ as a reasonable value that obtains a speedup of about 25-fold with a minimal loss of accuracy (Figure 3 and Table 1, row 3 compared to 13).

What is the best value to use for the S_{min} parameter, which specifies the minimum target survival fraction S during filter thresholding? Table 1 shows benchmark results for FST HMM filtering with $F = 0.993$ and three different S_{min} values (rows 14-16). We choose to set the default $S_{min} = 0.02$ because it gives a slightly lower MER than not enforcing an S_{min} (row 10) at about a 10% cost in running time. The effect of $S_{min} = 0.02$ can be seen in more detail in Tables 2 and 3. Table 2 shows that although enforcing S_{min} significantly reduces the speedup for families in which the FST determined S is less than 0.02, it has a small overall effect on the total speedup. Table 3 compares the speedup and filter sensitivity of using no S_{min} and using $S_{min} = 0.02$ for different final reporting thresholds, showing that although the time cost of enforcing $S_{min} = 0.02$ increases as the final threshold becomes more strict, the boost to sensitivity also

increases.

How much does FST calibrated HMM filtering impact sensitivity and specificity? Tables 1, 2 and 3 demonstrate FST's impact on benchmark performance. Table 2 shows that the actual sensitivity (actual F) achieved by the filter on our benchmark is 0.924. The summary and family MER for an HMM filtered search using $F = 0.993$ and $S_{min} = 0.02$ are 144 and 134 (Table 1 row 10 down from 130 and 109 for a non-filtered search (row 3).

How does using FST to determine filter thresholds compare to using a single target survival fraction S as a thresholding method? Figure 3 plots benchmark summary MER versus running time for different filtering strategies: FST with various F values and target S thresholding for various S values. Target S thresholding is faster than FST for achieving MER values down to about 160, but FST is faster if lower MERs are desired. Tables 1, 2, and 3 also compare FST with target survival fraction target S methods.

Is FST robust to a wide range of final E-value thresholds? With FST, the filter threshold increases as the final threshold increases (becomes more strict), increasing the filter's efficiency while theoretically maintaining the same level of sensitivity, F. Table 3 shows the effect of varying the final E-value threshold on the sensitivity and speed of FST calibrated HMM filters on the benchmark dataset. As E decreases, the sensitivity remains relatively constant while the speedup increases, until $E = 1e - 3$ is reached, at which point sensitivity begins to decrease, suggesting FST is less reliable for stricter thresholds. Fortunately, enforcing $S_{min} = 0.02$ corrects this problem. This is because many FST calibrated thresholds for final thresholds $E < 1e - 3$ correspond to $S < 0.02$, so enforcing S_{min} lowers the filter threshold and increases sensitivity.

What impact does the QDB CYK filtering approach have on speed, sensitivity and specificity? Rows 6-9 of Table 1 show benchmark performance using only a CYK filter with QDB and different tail loss β values. The filter thresholds were determined using a simple scheme, by setting the filter E-value threshold as 100 times the final E-value threshold. This thresholding strategy proved adaquate, using it with a CYK filter with $\beta = 10^{-10}$ results in about a four-fold speedup with a negligible loss in sensitivity relative to a non-filtered run (row 3). Further, this strategy yields significantly better performance than running non-filtered CYK with identical $\beta = 10^{-10}$ (row 5), while only requiring about 10% longer to run. This clearly suggests it is more useful to use QDB CYK as a filter for Inside than as the final scoring algorithm as we did previously [16].

Is it useful to combine a FST calibrated HMM filter and a QDB CYK filter? As mentioned above, FST

calibrated HMM filters with $F = 0.993$ and $S_{min} = 0.02$ result in about a 25-fold speedup and QDB CYK filters with $\beta = 10^{-10}$ result in about a four-fold speedup. Combining these two filtering strategies by running the HMM first, searching the surviving fraction with QDB CYK, and using Inside only on the fraction that survives both, results in about a three-fold speedup relative to only using HMM filters with a negligible loss of accuracy (compare rows 15 and 18 of Table 1. This strategy is about 70 times faster than the top performing strategy, non-filtered Inside search with $\beta = 10^-15$ at a small cost to sensitivity. And it is more than 200 times faster than non-banded Inside, while achieving a lower summary MER. Based on this, we've made this two filter strategy the default filtering strategy in INFERNAL version 1.01.

Discussion

FST is a general method for determining filter survival score thresholds to achieve a target level of sensitivity that can be applied to any database similarity search method. It is particularly easy to apply FST to search methods that use generative probabilistic models because the requisite test sequences for the threshold calibration can be sampled directly from the model. Here, we have explored the performance of FST on RNA similarity searches with CMs using this sampling technique, and show that on our benchmark, using HMM filters with FST calibrated thresholds reduces running time by 25-fold with a modest cost to sensitivity.

FST calibrated thresholds are query-dependent, and are most advantageous relative to query-independent thresholds, such as the target S thresholding method, for filtering methods in which different queries require significantly different surivival thresholds to achieve high sensitivity. We expect this is mostly true for filtering methods that score sequences in a qualitatively different way than the final search method, as in the case reported here which uses HMMs to filter, that score only primary sequence conservation, for CMs, which score both primary sequence and secondary structure conservation. However, when the filter scoring metric is more closely related to the final metric, simpler thresholding strategies, such as picking a single query independent threshold that empirically performs well on a benchmark may be more reasonable. This is the case with our experiments using the CM CYK algorithm as a filter for the CM Inside algorithm.

FST calibrated thresholds are also dependent on the reporting threshold of the final search method. This is demonstrated for CM filtering by the trajectory of the large, open circle points in Figure 1 that indicate filter threshold/final threshold pairs (T, C) pairs. As the CM reporting score threshold increases from a to b, the filter survival threshold necessary to maintain sensitivity also increases because the filter can now afford to miss hits in the score range a to b without affecting sensitivity. And as this survival threshold increases,

so does the acceleration gained from the filter. This feature of FST is useful for search methods where the reporting threshold chosen by users can vary widely for different applications. This is the case with CMs, where searches can range in magnitude from RFAM's annotation of the 120 Gb RFAMSEQ database, to searches in a prokaryotic genome of a few Mb. A reporting threshold of $E = 1$ in these two types of searches corresponds to significantly different bit scores because of the large size difference of the databases being searched, thus the survival reporting threshold will differ markedly between them, offering greater acceleration for the large RFAMSEQ search than for the prokaryotic genome search.

The slow speed of CM searches has been the most serious obstacle to the use of INFERNAL for annotating RNAs in databases and genomes. Without using filters, running the most sensitive CM search algorithm with INFERNAL version 1.01 required about 1500 hours to complete our benchmark search of 51 families against both strands of a 10 Mb database. We have shown that by combining FST calibrated HMM filters and QDB CYK filters with F, S_{min}, and β parameters that do not significantly compromise specificity or sensitivity, the running time drops 70-fold to about 20 hours. Eventually, we want to be able to use INFERNAL to annotate RNAs in large genomes in at most a few days. If our benchmark results hold for the general case, to run the 1371 RFAM release 9.1 families against the entire human genome would require about 20 CPU years (compared to 1500 CPU years for a non-filtered search), which means further acceleration remains an important goal of INFERNAL development.

We can imagine several ways to make INFERNAL faster. One is to use faster filters. A new version (3.0) of the HMMER software package is in it's last throes of development, and includes significantly faster HMM search algorithm implementations than those in INFERNAL 1.01. We plan to incorporate those implementations within INFERNAL for filtering. Other possible filtering strategies include using BLAST-like algorithms, or keyword based methods such as those described by Zhang et al. [21]. But the HMM filters are not the rate limiting step in CM searches, the time required to run the filter on our benchmark is about one third the total time of the search (Table 1 rows 18 and 19). So, unless we can design filters with lower survival fractions, the maximum acceleration we can gain from faster filters is about 33%. A complementary approach is to write faster implementations of the final CM search algorithms, Inside and CYK. Ongoing work on HMMER 3 has suggested that optimizing the dynamic programming search algorithm implementations using single-instruction multiple data (SIMD) paralellism could yield significant speedups. Developing these improvements – and incorporating them into a widely useful, freely available codebase – are priorities for us.

Acknowledgements

We thank Goran Ceric for his unparalleled skill in managing Janelia Farm's high performance computing resources.

Funding

EPN and SRE are supported by the Howard Hughes Medical Institute.

References

[1] T. M. Lowe and S. R. Eddy. tRNAscan-SE: A program for improved detection of transfer RNA genes in genomic sequence. *Nucl. Acids Res.*, 25:955–964, 1997.

[2] D. Laslett and B. Canback. ARAGORN, a program to detect tRNA genes and tmRNA genes in nucleotide sequences. *Nucl. Acids Res.*, 32:11–16, 2004.

[3] T. M. Lowe and S. R. Eddy. A computational screen for methylation guide snoRNAs in yeast. *Science*, 283:1168–1171, 1999.

[4] P. Schattner, S. Barberan-Soler, and T. M. Lowe. A computational screen for mammalian pseudouridylation guide H/ACA RNAs. *RNA*, 12:15–25, 2006.

[5] E. C. Lai, P. Tomancak, R. W. Williams, and G. M. Rubin. Computational identification of *Drosophila* microRNA genes. *Genome Biol.*, 4:R42, 2003.

[6] L. P. Lim, M. E. Glasner, S. Yekta, C. B. Burge, and D. P. Bartel. Vertebrate microRNA genes. *Science.*, 299:1540, 2003.

[7] T. J. Macke, D. J. Ecker, R. R. Gutell, D. Gautheret, D. A. Case, and R. Sampath. RNAMotif, an RNA secondary structure definition and search algorithm. *NAR*, 29:4724–4735, 2001.

[8] D. Gautheret and A. Lambert. Direct RNA motif definition and identification from multiple sequence alignments using secondary structure profiles. *J. Mol. Biol.*, 313:1003–1011, 2001.

[9] S. Zhang, B. Haas, E. Eskin, and V. Bafna. Searching genomes for noncoding RNA using FastR. *IEEE/ACM Trans. Comput. Biol. Bioinform.*, 2:366–379, 2005.

[10] Z. Huang, Y. Wu, J. Robertson, L. Feng, R. Malmberg, and L. Cai. Fast and accurate search for non-coding RNA pseudoknot structures in genomes. *Bioinformatics*, 24:2281–2287, 2008.

[11] Y. Sakakibara, M. Brown, R. Hughey, I. S. Mian, K. Sjölander, R. C. Underwood, and D. Haussler. Stochastic context-free grammars for tRNA modeling. *Nucl. Acids Res.*, 22:5112–5120, 1994.

[12] S. R. Eddy and R. Durbin. RNA sequence analysis using covariance models. *Nucl. Acids Res.*, 22:2079–2088, 1994.

[13] M. P. Brown. Small subunit ribosomal RNA modeling using stochastic context-free grammars. *Proc. Int. Conf. Intell. Syst. Mol. Biol.*, 8:57–66, 2000.

[14] R. Durbin, S. R. Eddy, A. Krogh, and G. J. Mitchison. *Biological Sequence Analysis: Probabilistic Models of Proteins and Nucleic Acids*. Cambridge University Press, Cambridge UK, 1998. ISBN 0521629713.

[15] S. R. Eddy. A memory-efficient dynamic programming algorithm for optimal alignment of a sequence to an RNA secondary structure. *BMC Bioinformatics*, 3:18, 2002.

[16] E. P. Nawrocki and S. R. Eddy. Query-dependent banding (QDB) for faster RNA similarity searches. *PLoS Comput. Biol.*, 3:e56, 2007.

[17] P. P. Gardner, J. Daub, J. G. Tate, E. P. Nawrocki, D. L. Kolbe, S. Lindgreen, A. C. Wilkinson, R. D. Finn, S. Griffiths-Jones, S. R. Eddy, and A. Bateman. Rfam: Updates to the RNA families database. *Nucl. Acids Res.*, 37:D136–D140, 2009.

[18] E. K. Freyhult, J. P. Bollback, and P. P. Gardner. Exploring genomic dark matter: A critical assessment of the performance of homology search methods on noncoding RNA. *Genome Res.*, 17:117–125, 2007.

[19] Z. Weinberg and W. L. Ruzzo. Exploiting conserved structure for faster annotation of non-coding RNAs without loss of accuracy. *Bioinformatics*, 20 Suppl. 1: I334–I341, 2004.

[20] Z. Weinberg and W. L. Ruzzo. Sequence-based heuristics for faster annotation of non-coding RNA families. *Bioinformatics*, 22:35–39, 2006.

[21] S. Zhang, I. Borovok, Y. Aharonowitz, R. Sharan, and V. Bafna. A sequence-based filtering method for ncRNA identification and its application to searching for riboswitch elements. *Bioinformatics*, 22:e557–e565, 2006.

[22] R. J. Klein and S. R. Eddy. RSEARCH: finding homologs of single structured RNA sequences. *BMC Bioinformatics*, 4:44, 2003.

[23] E. P. Nawrocki, D. L. Kolbe, and S. R. Eddy. Infernal 1.0: Inference of RNA alignments. Bioinformatics, in press, 2009.

[24] W. R. Pearson. Comparison of methods for searching protein sequence databases. *Protein Sci.*, 4:1145–1160, 1995.

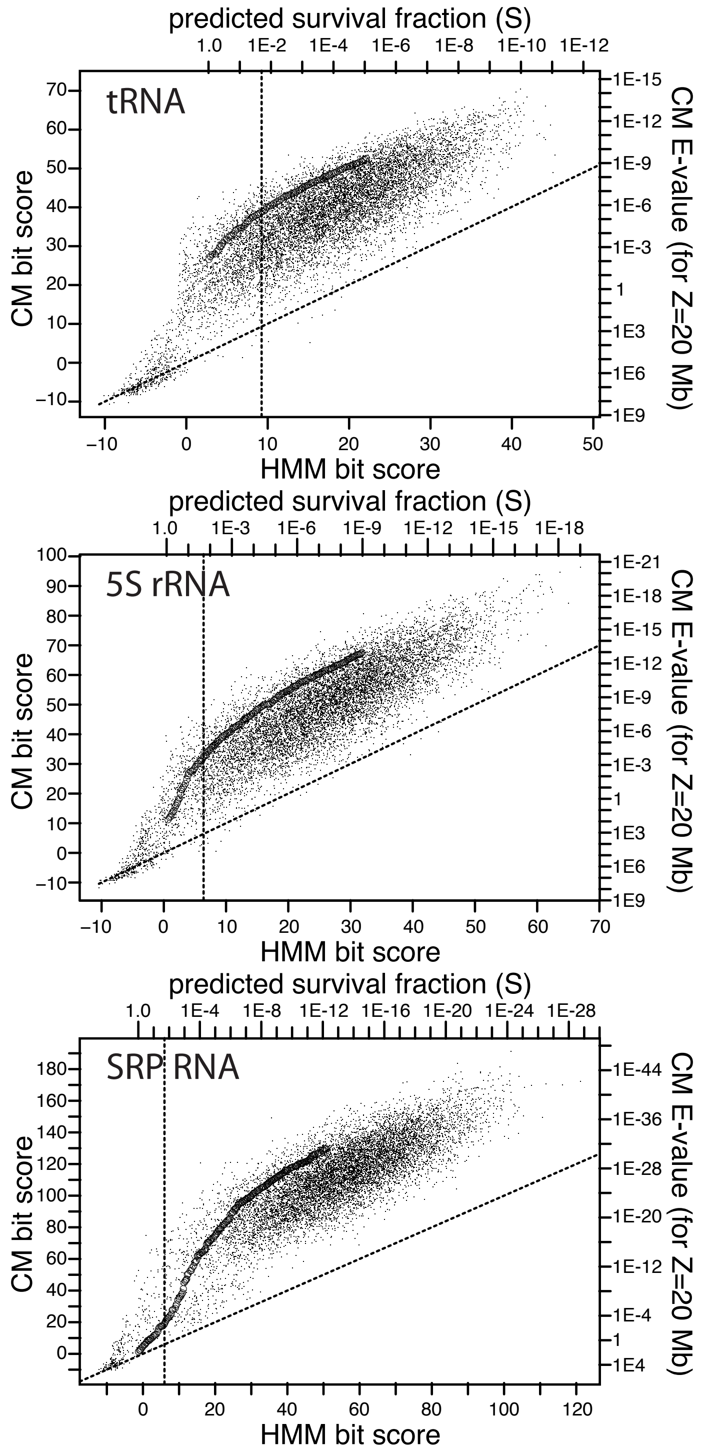

Figure 1: **CM Inside scores versus HMM Forward scores during FST calibration.** Complete data for the FST calibration with $N = 10,000$ and $F = 0.99$ of three anecdotal Rfam 9.1 families: 5S rRNA, tRNA, and RNase P (RF00001, RF00005, RF00011). Each sequence is represented as a black point with x-coordinate equal to it's HMM Forward score, and y-coordinate equal to it's CM Inside score. Red circles indicate the representative set of saved filter survival threshold T and CM reporting score threshold C pairs saved to the CM file and used to determine thresholds during searching. The CMs used for sampling and scoring, and HMMs used for scoring were locally configured.

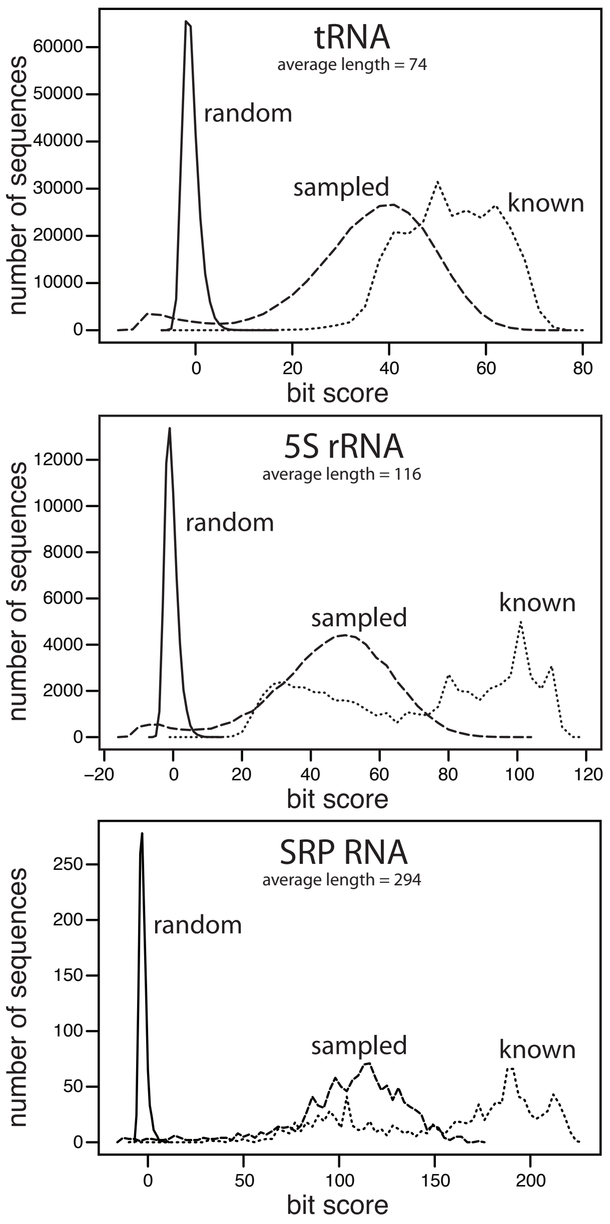

Figure 2: **CM score histograms of random, known, and sampled sequences for three RNA families.** CMs were built from RFAM 9.1 seed alignments using default parameters in INFERNAL 1.01 for three families: tRNA (RF00005), 5S rRNA (RF00001), and SRP RNA (RF00017). "random" sequences were generated independently for each family using a single state HMM with equiprobale emission probabilities (0.25) for the four possible RNA bases to be a specific length L, the average length of each family. The "sampled" sequences were sampled from locally configured CMs using the `cmemit` program of INFERNAL v1.01. The "known" sequences are the combination of the "seed" and "full" sequences from RFAM. All the sequences were scored using the non-banded Inside algorithm, and the scores were collated into a histogram of bit scores. The number of "random" and "sampled" sequences was set per family to be equal to the number of "known" sequences for that family: $261,247$ for tRNA, $57,766$ for 5S, and 1187 for SRP.

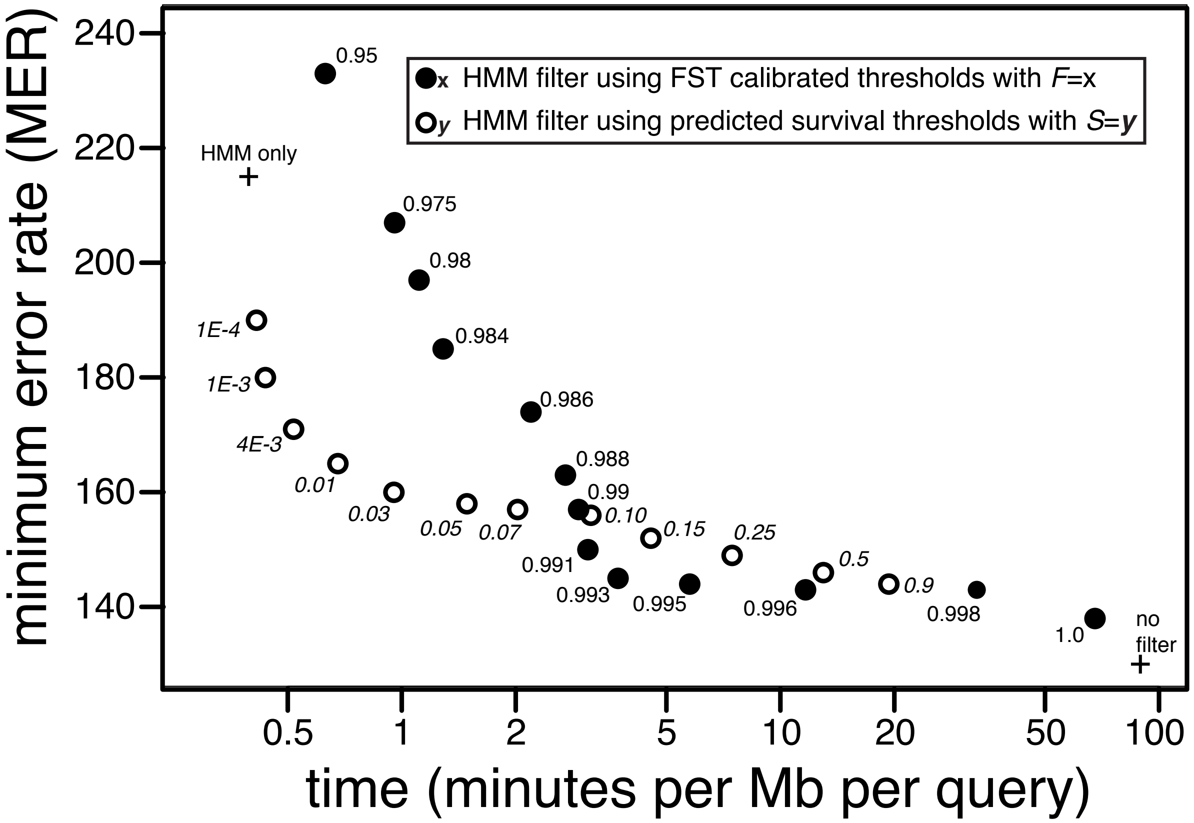

Figure 3: **MER versus time for the benchmark.** Solid black points show benchmark performance for HMM filtered searches using query-dependent FST calibrated filter thresholds with target sensitivity $F = x$, with x labelled per point. Open-circle points show benchmark performance for HMM filtered searches using a single, query-independent, target survival threshold of $S = y$, with y labelled per point. There are two additional "+" points: "HMM only": HMM Forward algorithm as the final scoring algorithm (with no filters); "no filter" Inside with QDB ($\beta = 10^{-15}$) as the final algorithm. For the FST searches $S_{min} = 0.$. All searches performed with INFENRAL 1.01. Note that the x-axis is in log-scale.

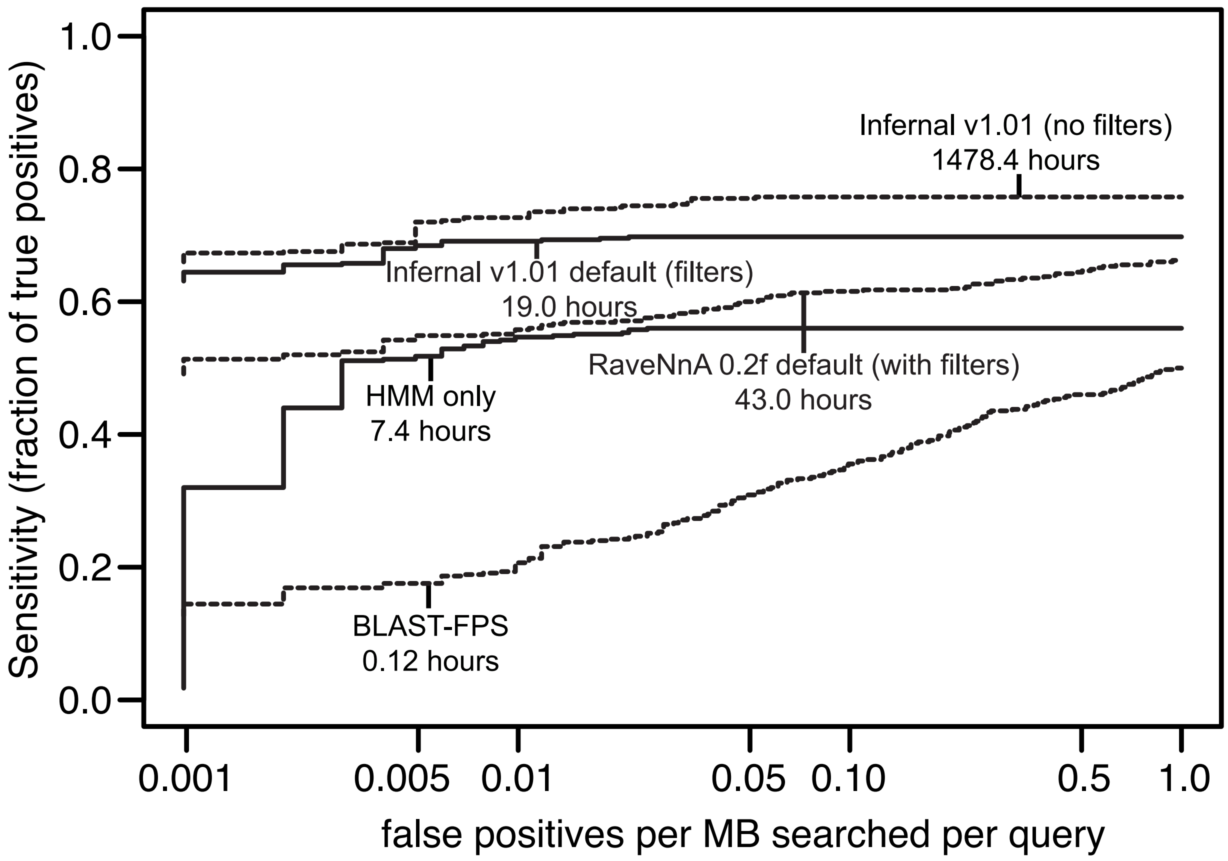

Figure 4: **ROC curves for the benchmark.** Plots are shown for INFERNAL 1.01 non-filtered CM searches, default filtered searches, and HMM only searches, and for RAVENNA 0.2f searches and for family-pairwise-searches (FPS) with BLASTN.

	filtering with HMM				filtering with CM		post-filtering		summary	family	time
	algorithm	FST F	S_{min}	target S	algorithm	QDB β	algorithm	QDB β	MER	MER	(min/Mb/query)
1	-	-	-	-	-	-	Inside	-	150	115	280.60
2	-	-	-	-	-	-	CYK	-	156	133	102.16
3	-	-	-	-	-	-	Inside	10^{-15}	130	109	89.13
4	-	-	-	-	-	-	CYK	10^{-15}	153	130	30.60
5	-	-	-	-	-	-	CYK	10^{-10}	154	132	21.97
6	-	-	-	-	CYK	10^{-13}	Inside	10^{-15}	131	114	30.08
7	-	-	-	-	CYK	10^{-10}	Inside	10^{-15}	130	114	24.24
8	-	-	-	-	CYK	10^{-7}	Inside	10^{-15}	134	118	17.42
9	-	-	-	-	CYK	10^{-4}	Inside	10^{-15}	142	127	10.18
10	Forward	-	-	0.02	-	-	Inside	10^{-15}	160	149	0.95
11	Forward	-	-	0.10	-	-	Inside	10^{-15}	156	142	3.16
12	Forward	-	-	0.25	-	-	Inside	10^{-15}	149	131	7.46
13	Forward	0.993	-	-	-	-	Inside	10^{-15}	145	135	3.73
14	Forward	0.993	0.01	-	-	-	Inside	10^{-15}	144	134	3.84
15	Forward	0.993	0.02	-	-	-	Inside	10^{-15}	143	133	3.99
16	Forward	0.993	0.10	-	-	-	Inside	10^{-15}	143	132	5.64
17	Forward	-	-	0.02	CYK	10^{-10}	Inside	10^{-10}	161	154	0.68
18	Forward	0.993	0.02	-	CYK	10^{-10}	Inside	10^{-15}	143	134	1.26
19	-	-	-	-	-	-	HMM Forward	-	214	204	0.39

Table 1: **Benchmark MER and timing statistics for different search strategies.** Each search strategy is defined by the algorithms and parameters used by zero, one or two filtering stages and a final post-filtering stage. Under "filtering with HMM": "algorithm" lists if an HMM filter is applied first ("Forward"), or not at all ("-"); "FST F" lists the target sensitivity F used for FST threshold calibration, or "-" if FST was not used; "S_{min}" is the minimum predicted survival fractions used to set filter thresholds (potentially overriding the FST calibrated thresholds); "target S" shows the single, target predicted survival fraction used for all modles in non-FST HMM filtering strategies. Under "filtering with CM": "algorithm" lists if a CM "CYK" filter is applied (only on the surviving subsequences from the HMM filter if one was used) or not at all ("-"), and "QDB β" lists the tail loss probability used to calculate bands for the algorithm. Under "post-filtering": "algorithm" lists the main algorithm used for scoring subsequences that survive the $<= 2$ filtering stages; "QDB β" lists the tail loss probability for the band calculation for the main algorithm. The sensitivity and specificity of each strategy is summarized by "summary MER" and "family MER" as explained in the text. Lower MERs are better. "min/Mb/query" list minutes per Mb (1,000,000 residues) of search space per query model used to search. The benchmark contains 51 query models and 20 Mb of search space (both strands of the 10 Mb pseudogenome) as explained in the text.

Predicted survival fraction (S) range for FST HMM filter ($F = 0.993$, no S_{min})			# query	# test	non-filtered # found	FST HMM filtering ($F = 0.993$, no S_{min})		FST HMM filtering ($F = 0.993$, $S_{min} = 0.02$)		Non-FST HMM filtering single threshold ($S = 0.02$)	
						actual F	speedup	actual F	speedup	actual F	speedup
(no filter)	S	$= 1.0$	2	52	43	1.000	1.0	1.000	1.0	0.581	70.9
$1.0 >$	S	$>= 0.1$	11	98	76	0.987	10.6	0.987	10.6	0.974	79.3
$0.1 >$	S	$>= 1e-2$	17	165	135	0.919	52.8	0.919	51.0	0.911	88.6
$1e-2 >$	S	$>= 1e-3$	8	54	48	0.854	150.8	0.854	72.1	0.854	80.9
$1e-3 >$	S	$>= 1e-4$	7	53	31	0.807	185.0	0.871	103.0	0.871	121.2
$1e-4 >$	S	$>= 1e-5$	4	6	4	1.000	90.4	1.000	57.8	1.000	67.6
$1e-5 >$	S	> 0	2	22	4	0.750	265.5	0.750	121.6	0.750	143.6
	all		51	450	341	0.924	23.9	0.930	22.3	0.871	93.4

Table 2: **Comparison of filter sensitivity and benchmark acceleration for queries with different FST predicted filter survival fractions.** The 51 query benchmark families were categorized based on the predicted survival fraction S of a FST filtered HMM benchmark search with final reporting threshold $E = 1$. FST was performed with $F = 0.993$ and no S_{min} value. Column 1 lists the survival fraction category; the first row "no filter $S = 1.0$" corresponds to queries for which FST indicates $S >= 1.0$ so the HMM filter is turned off. The next three columns list the number of query families ("# query"), total number of test sequences ("# test"), and number of the test sequences that the main algorithm scores with $E <= 1$ ("non-filtered # found"). The remaining six columns compare three filtering strategies: FST HMM filtering using $F = 0.993$ and no S_{min} value (this is row 10 in Table 1), FST HMM filtering with $F = 0.993$ and no $S_{min} = 0.02$ (row 12 in Table 1), and non-FST filtering setting thresholds that give a predicted $S = 0.02$ (row 14 in Table 1). For each strategy: "actual F" lists the filter sensitivity per category, the fraction of the test sequences the main algorithm scores $E <= 1$ that also pass the filter score threshold and survive the filter; "speedup" lists the per-category acceleration of a filtered search versus a non-filtered search in the benchmark. Only HMM filters were used (no CYK filters). The main algorithm used was Inside with QDBs calculated with $\beta = 10^{-15}$.

main algorithm E-value reporting threshold (database size = 20 Mb)	corresponding database size for $E = 1$ threshold	non-filtered # found	FST HMM filtering ($F = 0.993$, no S_{min})		FST HMM filtering ($F = 0.993$, $S_{min} = 0.02$)		Non-FST HMM filtering single threshold ($S = 0.02$)	
			actual F	speedup	actual F	speedup	actual F	speedup
$E = 1e-5$	2 Tb	250	0.880	108.5	0.984	66.3	0.980	89.0
$E = 1e-4$	200 Gb	268	0.892	91.9	0.974	60.8	0.966	89.0
$E = 1e-3$	20 Gb	285	0.902	73.1	0.965	53.6	0.940	89.0
$E = 1e-2$	2 Gb	298	0.920	38.2	0.960	32.5	0.920	89.0
$E = 1e-1$	200 Mb	324	0.926	29.2	0.954	26.1	0.904	89.0
$E = 1$	20 Mb	341	0.924	23.9	0.930	22.3	0.871	89.0
$E = 10$	2 Mb	355	0.913	15.4	0.916	14.8	0.851	89.0
$E = 100$	200 Kb	368	0.910	4.9	0.913	4.8	0.834	89.0
$E = 1000$	20 Kb	391	0.910	2.9	0.910	2.9	0.800	89.0

Table 3: **Comparison of filter sensitivity and benchmark acceleration for different main algorithm reporting E-value thresholds.** Column 1 lists E, the main algorithm reporting E-value threshold in the benchmark (20 Mb, two strands of a 10 Mb pseudogenome). Column 2 lists the database size in which a score with E-value E from column 1 corresponds to $E = 1$. Column 3 lists the number of the 450 test sequences the main algorithm scores with an E-value $< E$ from column 1. The remaining six columns compare the same three filtering strategies as in Table$\tilde{2}$ by filter sensitivity ("actual F") and acceleration of a filtered search versus a non-filtered search ("speedup"). Filter sensitivity is the fraction of test sequences the main algorithm scores with an E-value $< E$ from column 1 that also pass the filter score threshold and survive the filter. Only HMM filters were used (no CYK filters). The main algorithm used was Inside with QDBs calculated with $\beta = 10^{-15}$.
